# Repeated Dosing of Ketamine in the Forced Swim Test: Are Multiple Shots Better Than One?

**DOI:** 10.3389/fpsyt.2021.659052

**Published:** 2021-05-11

**Authors:** Ridge G. Weston, Paul J. Fitzgerald, Brendon O. Watson

**Affiliations:** Department of Psychiatry, University of Michigan, Ann Arbor, MI, United States

**Keywords:** chronic ketamine, forced swim test, major depression, literature search, sex differences, strain differences, sustained effects, subchronic

## Abstract

The anesthetic drug ketamine has been successfully repurposed as an antidepressant in human subjects. This represents a breakthrough for clinical psychopharmacology, because unlike monoaminergic antidepressants, ketamine has rapid onset, including in Major Depressive Disorder (MDD) that is resistant to conventional pharmacotherapy. This rapid therapeutic onset suggests a unique mechanism of action, which continues to be investigated in reverse translational studies in rodents. A large fraction of rodent and human studies of ketamine have focused on the effects of only a single administration of ketamine, which presents a problem because MDD is typically a persistent illness that may require ongoing treatment with this drug to prevent relapse. Here we review behavioral studies in rodents that used repeated dosing of ketamine in the forced swim test (FST), with an eye toward eventual mechanistic studies. A subset of these studies carried out additional experiments with only a single injection of ketamine for comparison, and several studies used chronic psychosocial stress, where stress is a known causative factor in some cases of MDD. We find that repeated ketamine can in some cases paradoxically produce *increases* in immobility in the FST, especially at high doses such as 50 or 100 mg/kg. Several studies however provide evidence that repeated dosing is more effective than a single dose at *decreasing* immobility, including behavioral effects that last longer. Collectively, this growing literature suggests that repeated dosing of ketamine has prominent depression-related effects in rodents, and further investigation may help optimize the use of this drug in humans experiencing MDD.

## Introduction

In the last two decades, the anesthetic drug *(R,S)*-ketamine (this racemic mixture is referred to as “ketamine” hereafter) has been successfully repurposed as an antidepressant in humans suffering from Major Depressive Disorder (MDD) (1–4). This has been a significant advance in clinical psychopharmacology because, unlike commonly used monoaminergic antidepressants such as selective serotonin reuptake inhibitors (SSRIs), ketamine has rapidly acting antidepressant properties, including in treatment resistant MDD (2, 4, 5). Monoaminergic antidepressants are also not therapeutically effective in all individuals, and can have significant side effects, creating demand for novel agents such as ketamine (6). The initial repurposing of ketamine was based on a foundation of rodent behavioral studies largely conducted in the 1990's, using a variety of compounds that, like ketamine, block glutamatergic NMDA receptors (7–11), although the detailed molecular mechanisms of ketamine (a non-competitive NMDA receptor antagonist) began being elucidated later (8, 12). Thus, a combination of preclinical and clinical studies, a large number of which continue to be carried out, has established that use of ketamine is an important pharmacological option for clinicians in the treatment of MDD.

Rodent neuroscience models offer an excellent opportunity to elucidate the neural network mechanisms of new or repurposed medications such as ketamine. This in turn would enable us to understand limbic circuitry itself in new ways. Many studies have focused on this and an ongoing debate, particularly in the rodent preclinical literature, concerns the precise molecular and circuit-based mechanisms through which ketamine produces its favorable effects (13–16). This ongoing debate includes investigating the efficacy of the R enantiomer of ketamine in a rat learned helplessness model (15), and also potentially dose-dependent sex differences in response to ketamine (16). It is not clear at this time whether the molecular or circuit-based mechanisms of single dose ketamine differ just in magnitude or instead qualitatively from those of repeated administration, but qualitative differences are possible (17).

To date most rodent studies have used only single doses of ketamine, whereas in clinical settings repeated dosing is increasingly used to treat MDD (18–20). These rodent studies typically administer a single, systemic injection of ketamine, and then monitor behavior in the acute period afterward, or 24 h or more later (14). It is important to measure behavior beyond the acute time window, such as at the 24 h point, since ketamine has acute dissociative-like properties that can result in hyperlocomotion (21, 22) and be confounded with immobility-related behavior in the FST. For these reasons, when studying any drug in the FST it is important to also measure locomotor activity in an assay such as the open field test, to gauge whether changes observed in the FST are confounded with generalized hyperactivity (or hypoactivity). The topic of dosing frequency of ketamine is of particular importance, since in clinical settings multiple doses are often given over the course of weeks to months in individuals with MDD (18–20, 23) to prevent relapse (19, 20). While understanding the effects of repeated dosing of ketamine is important clinically, this topic is only beginning to be addressed preclinically in rodent settings where neural mechanisms can be deciphered. We can then ask, why does ketamine initially produce favorable effects? And why do these favorable effects then typically fade? These questions can only be answered if we have effective and reliable behavioral models in rodents to allow us to subsequently delve deeper into neural mechanisms.

In this brief review, we investigate the behavioral effects of repeated dosing of ketamine in rodents, focusing on the widely used behavioral assay of antidepressant compounds, the forced swim test (FST) (24, 25). We focus on the FST since at this time there are only a limited number of studies on the behavioral effects of repeated dose ketamine and those using the FST are the most numerous. That said, this appears to be a growing literature, which we summarize below. Importantly, some of these studies use chronic psychosocial stress, such as chronic unpredictable stress (CUS) [also known as unpredictable chronic mild stress (UCMS)], to determine how this affects treatment with ketamine. This is crucial since chronic stress often triggers MDD (26, 27) and stress models in rodents are one of our primary means for studying mood-related brain circuits (28). The single injection ketamine literature in the FST provides conflicting evidence as to whether prior chronic stress exposure modulates the behavioral response to this drug, with some studies showing stress-sensitivity (28, 29) and a number of others not (16, 30). This topic remains to be adequately addressed in future studies that use repeated ketamine administration.

We should point out that in recent years, the FST has been increasingly criticized as not being a direct measure of depression-related behavior (31). For example, it has been suggested that immobility in the FST represents a passive coping strategy that is a behavioral adaptation to an inescapable acute stressor, rather than modeling depression-like behavior (32–34), or represents an extinction-like response (35). In this scenario, the FST may be more of a screening test for putative antidepressant compounds in response to an acute stressor, although questions remain regarding the utility of the FST for predicting response to non-monoaminergic, fast-acting glutamatergic agents (33, 35). For all of these reasons, future rodent studies aimed at investigating the putative antidepressant properties of ketamine (or other drugs) should consider including a battery of other behavioral tests [sucrose preference, novelty suppressed feeding, splash; as well as open field (to test generalized hyperactivity)] in addition to the FST. Repeated ketamine administration is only beginning to be investigated in these other tests (36–38).

## Literature Search Details

As recently as July 6, 2020, we conducted a literature search of PubMed using the following terms: ketamine + repeated/twice/subchronic/chronic + “forced swim”/“forced swimming.” We identified 24 relevant studies that used repeated administration of ketamine to mice or rats. These FST data are summarized in Table 1, and comprise immobility as a behavioral readout. In the studies described in Table 1, ketamine was injected from 2 to 30 times (and in one study was orally administered), over a period of up to 7 weeks, at a dose that varied from 0.1 to 100 mg/kg. The time delay between the final administration of ketamine and when the FST was conducted varied greatly, from 30 min to 2 months, which should be considered when interpreting acute versus sustained effects of this drug. A subset of these publications also used a *single* injection of ketamine for comparison with the multiple injections, and these data were also included in Table 1. During some of the experiments, chronic unpredictable stress (CUS) or unpredictable chronic mild stress (UCMS) was used, prior to the FST. The table consists of mice and rats of both sexes and various strains, ranging in age from adolescents to adults. The studies primarily used racemic (*R,S*)-ketamine, although one study (42) as noted in the table only used the (*S*)-ketamine enantiomer.

**Table 1:** Summary of repeated dosing of (*R,S*)-ketamine in the rodent forced swim test (FST).

**Publication**	**Species**	**Strain**	**Sex**	**Age at start**	**Dose (mg/kg)**	**# of repeats**	**Time delay**	**Stressor**	**Immobility**
Thelen et al. (39)	Mice	C57	M	8–12 wk	3	Once daily for 21 days	24 h	Unstr	No Ch
		C57	M	8–12 wk	5	Once daily for 21 days	24 h	Unstr	No Ch
		C57	M	8–12 wk	10	Once daily for 21 days	24 h	Unstr	Decr
		C57	F	8–12 wk	3	Once daily for 21 days	24 h	Unstr	No Ch
		C57	F	8–12 wk	5	Once daily for 21 days	24 h	Unstr	No Ch
		C57	F	8–12 wk	10	Once daily for 21 days	24 h	Unstr	Incr
Clarke et al. (40)	Mice	CD-1	M	8–10 wk	10	Single injection	1 h	Unstr	Decr
		CD-1	M	8–10 wk	10	Single injection	2, 5, or 8 days	Unstr	No Ch
		CD-1	M	8–10 wk	10	3 repeats over 2 weeks	2 days	Unstr	Decr
		CD-1	M	8–10 wk	10	3 repeats over 2 weeks	8 days	Unstr	Decr
Krimmel et al. (41)	Mice	CD-1	M	7 wk	10	Single injection	1 h	Unstr	Decr
		CD-1	M	7 wk	10	Single injection	24 h	Unstr	Decr
		CD-1	F	7 wk	10	Single injection	1 h	Unstr	Decr
		CD-1	F	7 wk	10	Single injection	24 h	Unstr	No Ch
		CD-1	M	7 wk	10	Every 2nd week for 4 weeks	1 h	Unstr	Decr
		CD-1	M	7 wk	10	Every 2nd week for 4 weeks	24 h	Unstr	Decr
		CD-1	F	7 wk	10	Every 2nd week for 4 weeks	1 h	Unstr	Decr
		CD-1	F	7 wk	10	Every 2nd week for 4 weeks	24 h	Unstr	No Ch
		CD-1	M	7 wk	10	Every 2nd week for 3 weeks	1 h	Unstr	Decr
		CD-1	M	7 wk	10	Every 2nd week for 3 weeks	24 h	Unstr	Decr
		CD-1	F	7 wk	10	Every 2nd week for 3 weeks	1 h	Unstr	No Ch
		CD-1	F	7 wk	10	Every 2nd week for 3 weeks	24 h	Unstr	No Ch
Neves et al. (42)	Mice	CF-1	M	Adult	30 (*S*-ket)	Single injection	24 h	Unstr	No Ch
		CF-1	M	Adult	30 (*S*-ket)	Once daily for 14 days	2 days	Unstr	Decr
		CF-1	M	Adult	30 (*S*-ket)	Once daily for 14 days	4 days	Unstr	No Ch
		CF-1	M	Adult	30 (*S*-ket)	Once daily for 14 days	8 days	Unstr	No Ch
		CF-1	M	Adult	30 (*S*-ket)	Once daily for 14 days	15 days	Unstr	No Ch
		CF-1	M	Adult	30 (*S*-ket)	Once daily for 14 days	22 days	Unstr	No Ch
		CF-1	M	Adult	30 (*S*-ket)	Once daily for 5 days	24 h	Unstr	No Ch
Kara et al. (43)	Mice	ICR	Mix	10–12 wk	5	Single injection	30 min	Unstr	No Ch
		ICR	Mix	10–12 wk	10	Single injection	30 min	Unstr	Decr
		ICR	Mix	10–12 wk	5	Once daily for 3 weeks	30 min	Unstr	No Ch
		ICR	Mix	10–12 wk	10	Once daily for 3 weeks	30 min	Unstr	No Ch
Suárez-Santiago et al. (38)	Mice	NIH	M	2 months	10	Once daily for 5 days	20 min	Unstr	Incr
Chatterjee et al. (44)	Mice	Swiss	M	Not stated	100	Single injection	24 h	Unstr	No Ch
		Swiss	M	Not stated	100	Once daily for 10 days	30 min	Unstr	Incr
		Swiss	M	Not stated	100	Once daily for 10 days	5 days	Unstr	Incr
		Swiss	M	Not stated	100	Once daily for 10 days	10 days	Unstr	Incr
Popik et al. (45)	Mice	Swiss	M	Not stated	1.25	Single injection	30 min, 2 weeks	Unstr	No Ch
		Swiss	M	Not stated	2.5	Single injection	30 min, 2 weeks	Unstr	No Ch
		Swiss	M	Not stated	5	Single injection	30 min, 2 weeks	Unstr	No Ch
		Swiss	M	Not stated	10	Single injection	30 min, 2 weeks	Unstr	No Ch
		Swiss	M	Not stated	50	Single injection	30 min, 2 weeks	Unstr	Decr
Singh et al. (46)	Mice	Swiss	M	Adult	100	Once daily for 10 days	24 h	Unstr	Incr
Hou et al. (47)	Mice	Sw-K	M	Not stated	25	Single injection	24 h	Unstr	No Ch
		Sw-K	M	Not stated	50	Single injection	24 h	Unstr	No Ch
		Sw-K	M	Not Stated	100	Single injection	24 h	Unstr	No Ch
		Sw-K	M	Not stated	25	Once daily for 7 days	24 h	Unstr	No Ch
		Sw-K	M	Not stated	50	Once daily for 7 days	24 h	Unstr	No Ch
		Sw-K	M	Not stated	100	Once daily for 7 days	24 h	Unstr	Incr
Owolabi et al. (48)	Mice	Albino	Mix	Adult	3	Single injection	5 min	Unstr	Decr
		Albino	Mix	Adult	15	Single injection	5 min	Unstr	Decr
		Albino	Mix	Adult	15	Once daily for 21 days	24 h	Unstr	Incr
Zhang et al. (49)	Rat	S-D	M	7 wk	10	Once daily for 3 days	15–17 h	UCMS	Decr
		S-D	M	7 wk	10	Once daily for 7 days	15–17 h	UCMS	Decr
		S-D	M	7 wk	10	Once every 3 days for 21 days	15–17 h	UCMS	Decr
		S-D	M	7 wk	10	Once every 7 days for 21 days	15–17 h	UCMS	Decr
Jiang et al. (50)	Rat	S-D	M	35–49 days	10	Once daily for 14 days	1 week	CUS	No Ch
		S-D	M	35–49 days	10	Once daily for 14 days	7 weeks	CUS	Decr
Li et al. (51)	Rat	S-D	M	28 days old	20	Once daily for 21 days	24 h	Unstr	No Ch
		S-D	M	28 days old	30	Once daily for 21 days	24 h	Unstr	No Ch
Parise et al. (36)	Rat	S-D	M	35–49 days	20	Single injection	1 h	CUS	Decr
		S-D	M	75–89 days	20	Twice daily for 15 days	2 months	CUS	Decr
		S-D	M	35–49 days	20	Twice daily for 15 days	2 months	CUS	Decr
		S-D	M	35–49 days	5	Twice in a day	24 h	Unstr	No Ch
		S-D	M	35–49 days	10	Twice in a day	24 h	Unstr	Decr
		S-D	M	35–49 days	20	Twice in a day	24 h	Unstr	Decr
Getachew and Tizabi (52)	Rat	Wist	M	8–10 wk	2.5 + alc	Once daily for 7 days	18 h	Unstr	Decr
Garcia et al. (53)	Rat	Wist	M	60 days	5	Once daily for 14 days	1 h	Unstr	Decr
		Wist	M	60 days	10	Once daily for 14 days	1 h	Unstr	Decr
		Wist	M	60 days	15	Once daily for 14 days	1 h	Unstr	Decr
Chindo et al. (54)	Rat	Wist	Mix	Adult	30	Single injection	24 h	Unstr	No Ch
		Wist	Mix	Adult	1	Once daily for 10 days	24 h, then weekly	Unstr	No Ch
		Wist	Mix	Adult	10	Once daily for 10 days	24 h, then weekly	Unstr	No Ch
		Wist	Mix	Adult	30	Once daily for 10 days	24 h, then weekly	Unstr	Incr
		Wist	Mix	Adult	50	Once daily for 10 days	24 h, then weekly	Unstr	Incr
De Cartágenes et al. (55)	Rat	Wist	F	35 days	10	Once daily for 3 days	3 h	Unstr	Incr
Réus et al. (56)	Rat	Wist	M	3 months	30	Once daily for 14 days	1 h	Unstr	Decr
Ecevitoglu et al. (57)	Rat	Wist	M	Adult	Up to 0.2/day	16 days in drinking water	24 h then 48 h	Unstr	No Ch
		Wist	M	Adult	Up to 0.4/day	16 days in drinking water	24 h then 48 h	Unstr	Decr
Popik et al. (45)	Rat	Wist	M	Not Stated	160	Single injection	6 days, 7 days	Unstr	No Ch
		Wist	M	Not Stated	50	Once daily for 2 days	40 min	Unstr	Decr
		Wist	M	Not Stated	50	Twice daily for 2 weeks	40 min	Unstr	Decr
Aricioglu et al. (58)	Rat	Wist	M	8–10 wk	10	Single injection	12 h	UCMS	No Ch
		Wist	M	8–10 wk	10	Once daily for 3 weeks	12 h	UCMS	Decr
Tizabi et al. (59)	Rat	Wist	F	Adult	0.5	Single injection	30 min	Unstr	No Ch
		Wist	F	Adult	2.5	Single injection	30 min	Unstr	No Ch
		Wist	F	Adult	5	Single injection	30 min	Unstr	No Ch
		WKY	F	Adult	0.5	Single injection	30 min	Unstr	No Ch
		WKY	F	Adult	2.5	Single injection	30 min	Unstr	Decr
		WKY	F	Adult	5	Single injection	30 min	Unstr	Decr
		Wist	F	Adult	0.5	Once daily for 10 days	20–22 h	Unstr	No Ch
		Wist	F	Adult	2.5	Once daily for 10 days	20–22 h	Unstr	No Ch
		Wist	F	Adult	5	Once daily for 10 days	20–22 h	Unstr	No Ch
		WKY	F	Adult	0.5	Once daily for 10 days	20–22 h	Unstr	No Ch
		WKY	F	Adult	2.5	Once daily for 10 days	20–22 h	Unstr	Decr
		WKY	F	Adult	5	Once daily for 10 days	20–22 h	Unstr	Decr
		WKY	F	Adult	2.5	Once daily for 10 days	1 week	Unstr	Decr
		WKY	F	Adult	2.5	Once daily for 10 days	2 weeks	Unstr	No Ch
Akinfiresoye and Tizabi (60)	Rat	WKY	M	Adult	0.25	Once daily for 11 days	20 min	Unstr	No Ch
		WKY	M	Adult	0.5	Once daily for 11 days	20 min	Unstr	Decr

*This table comprises the 24 mice and rat studies from our literature search that used repeated administration of ketamine in the FST. The “Immobility” column in table indicates whether ketamine decreased or increased this measure relative to vehicle-injected animals subjected to the same stress condition (i.e., stressed or unstressed), in a statistically significant manner where p < 0.05. The “Time Delay” column represents the amount of time between the last (or only) administration of ketamine and when the FST was carried out. All experiments that used a single injection are marked in yellow, and those that used chronic stress are marked in red. Experiments that showed a statistically significant decrease in immobility are marked in green, whereas those with a significant increase are marked in blue. C57, C57BL/6J; Sw-K, Swiss-Kunming; S-D, Sprague-Dawley; Wist, Wistar; M, male; F, female; Mix, males and females; wk, weeks; S-ket, (S)-ketamine; alc, alcohol; Unstr, unstressed; Decr, decreased; Incr, increased; No Ch, no change; UCMS, unpredictable chronic mild stress; CUS, chronic unpredictable stress*.

## Summary of Literature

While the literature on repeated ketamine administration in the FST is somewhat limited at this time, we summarize these findings in Table 1. This table may already yield several principles or themes. One principle is that when the effects of ketamine are acutely measured in the FST (within an hour of final administration), whether after only a single injection or multiple injections, this drug tends to decrease immobility [for example: (41, 53)]. However, as noted above, this time window comprises the acutely intoxicating or dissociative-like effects of ketamine, and one remarkable aspect of this drug is its capacity to affect brain and behavior for hours or days *after* it has been largely systemically eliminated *in vivo* (in about 4 h). Therefore, we focus on the time period in the FST beginning 24 h or later after the last drug administration (14). While a number of the studies listed in Table 1 investigated acute effects of ketamine, many examined longer time delays after drug injection.

Inspection of Table 1 also reveals that not many studies of repeated ketamine have used chronic stress, even though as described earlier chronic stress or trauma is an etiological factor in human MDD (26, 27). While we identified only four studies (all carried out in rats) that used chronic stress in Table 1, in each of the experiments carried out there was a decrease in immobility, after either a single injection or multiple injections, that was statistically significant in most cases and lasted up to 2 months for repeated injections (36, 49, 50, 58). These daily unpredictable stress procedures varied in length from 15 (36) to 42 days (50, 58), and used either one (58) or two (36, 49, 50) stressors per day, such as cage tilting or placing wet bedding in the cage. These behavioral findings after chronic stress was administered are consistent with experiments from our laboratory that used C57BL/6J mice, and found that chronic stress was either necessary for (28) or amplified (29) the increases in swimming or decreases in immobility produced by a single injection of ketamine. Future studies should further investigate how repeated ketamine modulates the behavioral and neural effects of chronic stress.

In comparing mice with rats in Table 1: while studies often used different parameters such as varied dosing and none of the mouse studies used chronic stress, we can conclude that repeated ketamine is capable of producing either decreases or increases in immobility in the FST in both species, under various experimental conditions, with higher doses such 50 or 100 mg/kg favoring immobility. Regarding different strains of mice or rats: the three studies that used CD-1 and CF-1 mice only found decreases in immobility (40–42), whereas studies of other albino mouse strains and C57BL/6J mice showed either increases or decreases in immobility depending on the study parameters and strain used (39, 44–48). Likewise, Sprague-Dawley or WKY rats were only observed to have decreases in immobility (36, 49–51, 59, 60). One of these studies directly compared female Wistar and WKY rats, and found that the latter strain was more responsive to the immobility decreasing effects of this drug (59). As mentioned above, Neves et al. (42) was the only study in Table 1 that used the enantiomer, (*S*)-ketamine. It is noteworthy that this study showed an advantage of repeated over single dose ketamine at 30 mg/kg administered daily for 2 weeks, although the favorable effect of repeated drug did not persist beyond 2 days (42).

These preliminary observations on species and strain should be tested in further studies in a controlled manner. It is not clear whether these differences in the FST reflect differential response to ketamine or qualitatively different engagement by the strains with the FST itself. For example, in one strain, higher mobility may indicate more motivation or exploratory drive, but in another it may indicate depressive-like behavior that resembles psychomotor agitation—-which are on opposing ends of the typical spectrum of neuropsychiatric health states. Some of our own recent data suggest that male C57BL/6J mice, for example, differ in FST behavior from other common inbred and outbred strains, perhaps showing greater sensitivity to the immobility enhancing effects of one injection of 10 mg/kg ketamine in unstressed animals (14). We have also recently demonstrated that female C57BL/6J mice can be more sensitive than males to the favorable effects of a single dose of 30 mg/kg ketamine in the FST, an effect that is amplified by prior exposure to chronic stress (29). Finally, there is growing evidence that, in addition to factors such as animal strain, sex, and age, the sex of the experimenter may play a prominent role in the outcomes of behavioral experiments such as the FST (61, 62). These factors need to be considered in future studies that investigate repeated dosing of ketamine and other drugs.

Most of the experiments in Table 1 used male rather than female mice and rats. Inspection of the table reveals that both male and female mice and rats are capable of exhibiting either increases or decreases in immobility in response to repeated ketamine under different experimental conditions. For example, in females repeated doses as low as 10 mg/kg can produce *increases* in immobility (39, 55). There appears to be only a limited literature directly comparing males and females after repeated dosing of ketamine in the FST, although studies such as Thelen et al. (39) and Krimmel et al. (41) suggest that female mice may be less sensitive than males to the immobility decreasing effects of repeated ketamine. Several studies in the single injection ketamine literature suggest that female mice or rats show decreased immobility after lower doses (such as 10 mg/kg or less), but the duration of action may be longer in males, although our recent study may be an exception to this latter point (29, 63–65).

Is there an optimal dose for repeated ketamine that emerges from these studies? High doses, such as 50 or 100 mg/kg, can be associated with *increases* in immobility in Table 1 both acutely and up to 10 days later (44, 46, 54), although 50 mg/kg is still a subanesthetic dose. While we have previously suggested (14) that the field has converged on using 10 mg/kg as a standard dose in single injection rodent FST experiments (16), our own data have suggested that 30 mg/kg is more effective at reducing immobility in male C57BL/6J mice (28). A repeated dose as low as 10 mg/kg in Table 1 has actually been associated with increases in immobility in mice (38, 39, 55). The field has not converged on a single repeated dose found to be generally efficacious in all labs and contradictory results are frequent. This will encumber efforts to consistently study these phenomena. The variety of results may stem from strain differences (including between inbred versions of the same strain from different vendors or laboratories), handling differences (both by laboratory staff and animal housing staff), environmental differences, or differences in the testing and quantification itself. These problems are not new to behavioral pharmacology, but ketamine research has not avoided them.

Lastly and perhaps most importantly, are multiple administrations of ketamine more effective in the FST than a single administration of this drug, based on the limited data set described in Table 1? As stated above, chronic treatment with ketamine can increase immobility in some studies, especially at moderate to high doses (30–100 mg/kg). Whether repeated use of lower doses is more effective at reducing immobility than a single administration of that same dose or a different one, seems to be unclear at this time. Some studies that used both single and repeated injections show an advantage for chronic dosing over a single dose (40, 42, 58), whereas others show the opposite pattern (47, 54) or no clear advantage for either dosing regimen (41). The Chindo et al. (54) and Hou et al. (47) studies found immobility increasing effects of repeated ketamine at relatively high doses of this drug (30–100 mg/kg), which may suggest that repeated dosing can be more favorable than a single dose if a sufficiently low dose (such as 10 mg/kg or lower) is used repeatedly, or perhaps if there is a longer interval between administrations. Beyond there being some degree of difference in dose used in these above five studies, it is difficult to draw further conclusions from them on the efficacy of repeated dosing due to different experimental parameters used, such as (*S*)-ketamine (42) or chronic stress (58). In general, the scarcity of studies reviewed here that have used repeated ketamine in the FST precludes definitive conclusions on the potential interactions between variables such as strain, sex, stress, and dose. To better compare a single dose with repeated dosing, future studies should keep all other experimental variables the same, including the time lag between the last (or only) dose of ketamine and the start of the FST or other behavioral tests [sucrose preference, novelty suppressed feeding, splash; as well as open field (to test generalized hyperactivity)]. It should be noted that in a recent study using rats selectively bred for depressive-like behavior, repeated ketamine (10 mg/kg/day for 7 consecutive days) did not produce favorable brain network topological effects relative to vehicle or a single ketamine injection, calling into question the general approach of using repeated ketamine in this animal model (17).

A related topic is whether the immobility decreasing effects of repeated ketamine last longer than a single dose. For example, studies such as Clarke et al. (40) and Neves et al. (42), which directly compare in mice a single injection vs. multiple injections at the same dose, suggest that the immobility decreasing effects of repeated ketamine may last longer than those of one injection. Another point is that repeated dosing regimens are capable of producing a decrease in immobility when ketamine is given at different fixed intervals, including less frequently than once a day (40, 41, 49), exactly once a day (39, 42, 49, 50, 52, 53, 58–60), or twice a day (36). In Sprague-Dawley rats weekly administration of ketamine, for 5 weeks at 20 or 50 mg/kg, has previously been shown to induce locomotor sensitization (66). All of the experiments in Table 1 that repeatedly administered ketamine used a fixed interval. It would also be informative to study this drug when given at a variable interval. One possibility is that a variable interval may be less likely to produce mood cycling or therapeutic habituation, since drug administration would not occur at a fixed, repeating interval.

Future reverse translational studies of ketamine in rodents may benefit from investigating dosing regimens, such as three times a week for 2 weeks and related protocols, that have been used effectively in human MDD studies (1, 19, 67, 68). Another consideration in future rodent studies, as well as clinical trials, would be to test whether monoaminergic antidepressants can amplify or further sustain the antidepressant properties of ketamine (69), including the combination of esketamine with SSRIs or SNRIs in rodents or treatment resistant human subjects (70). Likewise, if treatment with a monoaminergic antidepressant is initiated but only results in a weak response for a given individual, perhaps ketamine can then be added to amplify the response. It has already been demonstrated in MDD that repeated intranasal esketamine plus a conventional antidepressant can be more effective than an antidepressant alone (71). Several studies in the rodent literature also suggest that SSRIs modulate the behavioral effects of ketamine. For example, chronically administered citalopram can hasten and sustain the anti-immobility effect of repeated ketamine (10 mg/kg once every 7 days for 3 weeks) (49), although another study that gave daily ketamine for 2 weeks at a higher dose (30 mg/kg/day) found no effect of a single dose of fluoxetine (72). A study that reported an *increase* in immobility with 30 mg/kg/day ketamine for 5 days showed that a single dose of paroxetine could attenuate this effect (54). One or two injections of sertraline can also counteract hyperlocomotion induced by 10 consecutive days of 15 mg/kg ketamine (73). Combining repeated ketamine with conventional monoaminergic antidepressants is a promising line of inquiry that should be investigated further.

Another approach has been suggested for prolonging the antidepressant properties of a single administration of ketamine: using (*R*)-ketamine instead of the racemic mixture of this drug or (*S*)-ketamine. A number of rodent studies that used a variety of depression-related tests including the FST, have suggested that a single administration of (*R*)-ketamine has greater therapeutic potency and more sustained effects than (*S*)-ketamine (74–76). These studies also suggest that (*R*)-ketamine has fewer side effects, such as dissociative-like properties and hyperlocomotion, than (*S*)-ketamine. A body of repeated dosing studies in rodents also suggests that (*R*)-ketamine has more favorable effects on: bone mineral density in ovariectomized mice (77), MPTP-induced dopaminergic neurotoxicity (78), and phencyclidine-induced cognitive deficits (79).

## Conclusions

Based on our literature search, we find that administering ketamine multiple times has prominent effects in the rodent FST. Perhaps most importantly, several studies that directly compared single with multiple injections of ketamine found evidence for greater efficacy at decreasing immobility, that is possibly of longer duration, with repeated drug administration, especially when lower doses (10 mg/kg or lower) are used. Further, repeated injections of this drug are capable of producing both increases in immobility (especially at higher doses such as 30–100 mg/kg) and decreases, both acutely and in a more sustained fashion. Within the 1st h after administration, ketamine more reliably reduces immobility, but this may be confounded with dissociative-related hyperactivity. In the few studies that used chronic psychosocial stress, either single or repeated ketamine tended to reduce immobility. Both mice and rats (including males and females) are capable of exhibiting either increases or decreases in immobility after repeated ketamine (depending on experimental parameters such as dose or administration interval), and there is some evidence that this could differ by strain. Two studies suggest that female mice are less sensitive than males to the favorable effects of this drug in the FST, which is an important topic with respect to higher rates of MDD in women. Future studies should further investigate the optimal dosing interval (and dose) of repeated ketamine treatment in the rodent FST and related tests. The potential sex differences in response to ketamine have been particularly understudied at this time, and we are also only beginning to appreciate whether certain strains such as C57BL/6J mice are better than others for modeling depression-related behavior and its response to ketamine (14). These future studies should be conducted with an eye toward maximizing the sustained antidepressant effects of this unique pharmacological agent in the treatment of MDD in human subjects.

## Author Contributions

RW, PF, and BW conceived of this review, wrote, and edited the manuscript. RW performed the literature search and constructed the table, with assistance from PF. All authors contributed to the article and approved the submitted version.

## Conflict of Interest

The authors declare that the research was conducted in the absence of any commercial or financial relationships that could be construed as a potential conflict of interest.
